# Nano-Engineered Delivery of the Pro-Apoptotic KLA Peptide: Strategies, Synergies, and Future Directions

**DOI:** 10.3390/biom16010074

**Published:** 2026-01-02

**Authors:** Yunmi Cho, Ha Gyeong Kim, Eun-Taex Oh

**Affiliations:** Department of Biomedical Sciences, College of Medicine, Inha University, Incheon 22212, Republic of Korea; yjm10151015@naver.com (Y.C.); hagyeong315@naver.com (H.G.K.)

**Keywords:** KLA peptide, cancer, nano-engineered delivery

## Abstract

Antimicrobial peptides have been increasingly recognized as potential anticancer agents, with the KLA peptide (KLAKLAK_2_) being one of the most well-known and successful examples. The research interest in the KLA peptide is attributed to its ability to induce apoptosis in cancer cells by disrupting the mitochondrial membrane. However, the KLA peptide exhibits poor cellular uptake and it lacks targeting specificity, limiting its clinical potential in cancer therapy. In this review, recent advances in nano-engineered delivery platforms for overcoming the limitations of KLA peptides and enhancing their anticancer efficacy are discussed. Specifically, various nanocarrier systems that enable targeted delivery, controlled release and/or improved bioavailability, including pH-responsive nanosystems, photo-chemo combination liposomes, self-assembled peptide-based nanostructures, nanogel-based delivery systems, homing domain-conjugated KLA structures, inorganic-based nanoparticles, and biomimetic nanocarriers, are highlighted. Additionally, synergistic strategies for combining KLA with chemotherapeutic agents or immunotherapeutic agents to overcome resistance mechanisms in cancer cells are examined. Finally, key challenges for the clinical application of these nanotechnologies are summarized and future directions are proposed.

## 1. Introduction

Cancer is a leading cause of death worldwide, with approximately 20 million new cases and 9.7 million deaths annually [1]. Despite significant advances in chemotherapy, clinical outcomes remain limited in a significant number of patients due to systemic toxicity and drug resistance [2,3]. Evasion of apoptosis, particularly through dysregulation of mitochondrial-dependent pathways, is a hallmark of cancer and is observed in a wide range of malignancies, making it an important therapeutic target [4,5].

The canonical KLA peptide consists of 14 amino acids arranged as two repeats of the KLAKLA motif, a sequence organization that is critical for its biological activity [6,7,8]. Although extended variants such as the 21-mer KLAKLAK_3_ have also been investigated to modulate structural stability and biological activity, this repetitive arrangement promotes the formation of a stable amphipathic α-helical secondary structure composed of alternating positively charged lysine (K) residues and hydrophobic amino acids such as leucine (L) or alanine (A) [6].

The amphipathic α-helical conformation of the KLA peptide is defined by a distinct spatial segregation of hydrophilic, positively charged residues and hydrophobic residues, resulting in clearly separated polar and nonpolar faces [6]. This physicochemical feature facilitates preferential interaction with negatively charged phospholipids on cellular membranes [9]. Following cellular uptake, the KLA peptide predominantly accumulates at the mitochondrial outer membrane, where it disrupts the mitochondrial potential (ΔΨm) and induces mitochondrial outer membrane permeabilization [10]. As a result, cytochrome c is released into the cytoplasm, activating the caspase cascade and initiating the intrinsic apoptosis pathway [10,11]. Based on these mechanisms, the KLA peptide has been characterized as a novel apoptosis-inducing agent that directly targets mitochondria, which is a key organelle that determines intracellular energy metabolism and survival [12].

**Figure 1:**
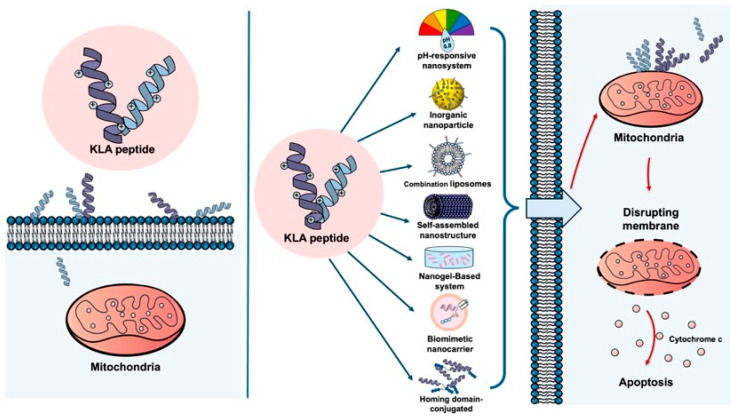
KLA peptide delivery with and without nano-engineered carriers. (**Left**) Free KLA peptide shows poor cellular uptake and limited mitochondrial targeting in cancer cells, resulting in minimal apoptotic activity. (**Right**) Nano-engineered carriers enhance cellular uptake of the KLA peptide in cancer cells, promote mitochondrial localization, and induce mitochondrial disruption and apoptosis. Created using Microsoft PowerPoint (Office 365).

The ability of the KLA peptide to cause cancer cell death by destroying mitochondria has been increasingly recognized as an anticancer strategy [13]. It has been reported that cancer cell death can be induced by the KLA peptide, and synergistic effects have been observed when it is combined with conventional anticancer drugs [14] or radiotherapy [15]. However, several limitations surrounding the use of KLA peptides alone have been reported. These include low cell permeability, as noted above, which makes it difficult for KLA peptides to spontaneously cross cell membranes [6,16,17,18]; non-specific toxicity to normal cells [18]; and limited delivery within the tumor microenvironment [19]. Furthermore, KLA peptides are rapidly degraded by serum proteases and exhibit poor pharmacokinetic stability in vivo, further limiting their clinical applicability [20] (Figure 1).

Strategies to improve the intracellular delivery and tumor selectivity of the KLA peptide as well as enhance their anticancer efficacy, including homing domains, sequence modifications, and the addition of various functionalities, have been suggested in recent review [21]. The need to systematically summarize the latest nano-engineering approaches for KLA peptide delivery and identify the most effective therapeutic strategies has thus been highlighted.

To overcome these problems, a variety of nanotechnology-based delivery platforms have been reported, exemplified by pH-responsive nanosystems, photo-chemo combination liposomes, self-assembled peptide-based nanostructures, nanogel-based delivery systems, homing domain-conjugated KLA structures, inorganic-based nanoparticles and biomimetic nanocarriers, with a focus on the improvement of intracellular uptake, biostability, and tumor-targeting capabilities of KLA peptides for cancer therapy [18,21,22,23,24,25]. Consequently, nanotechnology-based approaches have been proposed as promising solutions, offering enhanced stability, targeted delivery, and synergistic therapeutic effects in cancer treatment.

In this review, recent advances in nano-engineered delivery designed to enhance the therapeutic potential of the pro-apoptotic KLA peptide for cancer treatment are summarized. Key design strategies for improving the stability, tumor selectivity, and mitochondrial-targeting efficiency of KLA peptides, as well as the synergistic effects achieved through combination therapy with conventional anticancer drugs and radiotherapy, are summarized. In addition, major challenges impeding clinical translation and potential future research directions are addressed. Current research achievements and future directions are integrated to provide insights into the development of next-generation KLA peptide delivery platforms for improved cancer therapy.

## 2. Nano-Engineering-Based Delivery Systems for the Pro-Apoptotic KLA Peptide: A Type-Specific Approach

Various nanotechnology-based delivery systems have been developed for the effective delivery of KLA peptides, each characterized by distinct nanostructural features and delivery mechanisms that are exploited to enhance cellular uptake, mitochondrial accessibility, and tumor selectivity. In this section, the design strategies and structural characteristics of representative platforms, including pH-responsive nanosystems, photo-chemo combination liposomes, self-assembled peptide-based nanostructures, nanogel-based delivery systems, homing domain-conjugated KLA structures, inorganic-based nanoparticles, and biomimetic nanocarriers, are systematically summarized. In addition, the relationship between delivery platform architecture and its contribution to improving the anticancer efficacy of KLA peptides is discussed.

### 2.1. pH-Responsive Nanosystems

pH-responsive nanosystems are designed to remain stable in normal tissues while being activated in the acidic tumor microenvironment or within intracellular organelles, thereby enabling controlled release of KLA peptides and co-delivered drugs for tumor-selective therapy.

**Figure 2:**
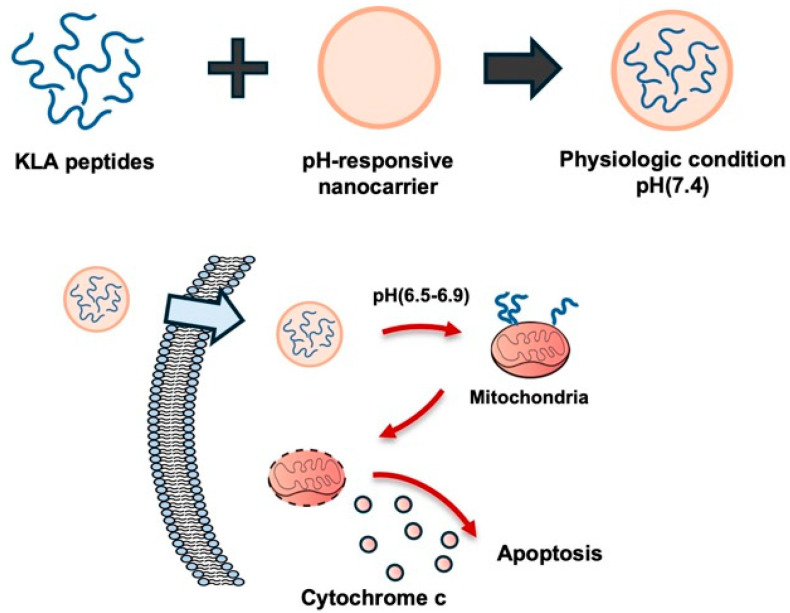
pH-responsive nanocarrier for tumor-selective KLA peptide delivery. The nanocarrier remains stable at physiological pH but becomes activated in the acidic tumor microenvironment, releasing the pro-apoptotic KLA peptide. The release of peptide localizes mitochondria and induces apoptosis in cancer cells. Created using Microsoft PowerPoint (Office 365).

The pH of the solid tumor microenvironment is acidic (pH 6.5–6.9) due to increased fermentative metabolism and poor perfusion compared with normal tissue under physiological extracellular pH (pHe) conditions (pHe 7.2–7.4) [26]. pH-responsive nanosystems are designed to remain stable in normal tissues but are activated by the acidic conditions within tumor tissues or endosomes/lysosomes within cells, releasing drugs or peptides [7,27]. In particular, pro-apoptotic KLA peptides target intracellular mitochondria, inducing ΔΨm collapse and apoptosis, allowing these pH-responsive transporters to enhance intracellular and mitochondrial accessibility [7,27] (Figure 2).

For example, a pH-responsive complex composed of glycol chitosan and dimethylaminopropyl methacrylate (DMA, 2,3-dimethylmaleic acid) was used for the delivery of the D-(KLAKLAK)_2_ peptide, exhibiting stability at pH 7.4 and charge reversal at pH 6.8, which enabled preferential release into tumor cells [27]. Furthermore, an amphiphilic poly(β-amino ester)-poly(ethylene glycol) was conjugated with the KLA peptide to generate CGKRK_D_(KLAKLAK)_2_. Micellar structures were formed at physiological pH (7.4), with docetaxel (DTX) encapsulated in the core. Mitochondrial delivery of _D_(KLAKLAK)_2_ was achieved, leading to mitochondria-dependent apoptosis in breast cancer cells. Co-delivered DTX contributed to microtubule disruption and cell cycle arrest, resulting in synergistic apoptotic effects in the cancer cells [7].

These pH-responsive nanocarriers have demonstrated advantages including reduced nonspecific toxicity to normal cells and enhanced tumor targeting and apoptosis induction. However, the complexity and difficulty of their synthesis and issues associated with scaling up their production remain significant challenges [28]. To overcome the limitations of single-stimulus systems, increasing attention has been directed toward multi-stimuli-responsive nanocarriers capable of responding to various intracellular and extracellular stimuli, including pH, temperature, redox potential, and light [29]. The integration of pH-sensitive and multi-responsive systems is expected to enhance tumor-selective delivery and the cell-killing efficiency of KLA peptides.

Enhanced cellular uptake and mitochondrial accessibility of KLA peptides can be achieved through these systems, resulting in tumor-selective anticancer effects; however, challenges related to synthetic complexity and scalable manufacturing remain.

### 2.2. Photo-Chemo Combination Liposomes

Photo-chemo combination liposomes are designed to incorporate photosensitizers that enable localized reactive oxygen species generation, which acts synergistically with the pro-apoptotic KLA peptide to induce tumor cell death.

Cancer treatments have traditionally included chemotherapy, radiation therapy and surgery, and more recently, immunotherapy [30]. However, chemotherapy often produces systemic side effects, surgery is associated with a high recurrence rate, and radiation therapy is limited by the cumulative radiation dose [30]. To improve treatment efficacy, photodynamic therapy (PDT) has been explored as a treatment modality capable of tumor removal while preserving normal tissue function [31]. First introduced in the early 1900s and demonstrated in its modern form by Dougherty et al. in 1975 [31], PDT, which has been extensively investigated, is recognized as a disease-site-specific treatment modality [30].

PDT is based on the principle of administering a photosensitizer that selectively accumulates in the tumor area, followed by irradiation of the area with light of a specific wavelength to activate the photosensor [32]. Activated photosensors transfer energy to molecular oxygen (O_2_), generating cytotoxic reactive oxygen species (ROS), particularly singlet oxygen (^1^O_2_)—a therapeutic strategy that results in tumor cell ablation through oxidation of key cellular macromolecules [32]. However, PDT has limited light-penetration depth [32], and its oxygen-dependence restricts its therapeutic efficacy in hypoxic tumor environments [33,34].

To overcome these limitations, photo-chemo combination strategies integrating chemodynamic therapy (CDT) with PDT have been proposed [35]. The combination of PDT and CDT has been shown to enhance ROS generation and activity [35]. H_2_O_2_ generated during PDT can be utilized as a substrate for CDT, facilitating the production of highly reactive ·OH radicals [35].

**Figure 3:**
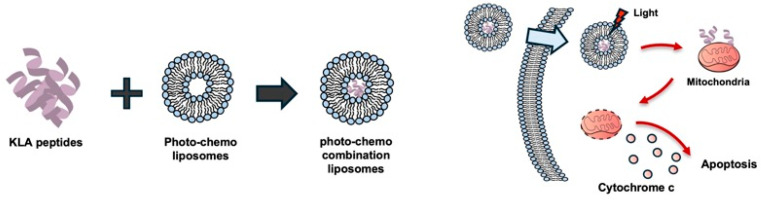
Photo-chemo combination liposomes for KLA peptide delivery. Liposomes co-loaded with KLA peptides and a photosensitizer generate reactive oxygen species upon light irradiation, facilitating endosomal membrane disruption together with the membrane-lytic activity of KLA peptides. Following endosomal escape, released KLA peptides localize to mitochondria and induce mitochondrial dysfunction and apoptosis in cancer cells. Created using Microsoft PowerPoint (Office 365).

Liposomes have been widely utilized as drug carriers and effect enhancers in PDT [36], and a liposome-based photo-chemo complex incorporating the KLA peptide has been introduced [37] (Figure 3). Specifically, a liposome complex system, termed Lipo[Pep, Ce6], was constructed, consisting of chlorin e6-conjugated di-block copolymer (PEG-PLL[-*g*-Ce6]) and D-(KLAKLAK)_2_ peptide-loaded liposomes (Lipo [PeP]) [37].

Because of the membrane-lytic ability of D-(KLAKLAK)_2_ and the membrane-disrupting effect of ^1^O_2_ generated from e6, Lipo[Pep, Ce6] accelerated the destruction of endosomal compartments and showed potent synergistic anticancer activity in vitro by virtue of the mitochondrial-disrupting effect of D-(KLAKLAK)_2_ [37].

PDT using photosensitizers has limitations, such as difficulties in deep tissue penetration of light and therapeutic efficacy in hypoxic tumor microenvironment due to the oxygen dependence of the PS [38,39]. Modifications to liposome composition, enhanced selective photosensitizer-accumulation efficiency, and improved deep tumor penetration using near-infrared light may contribute to overcoming these limitations and facilitating the clinical translation of KLA-peptide-based photo-chemo nanotherapeutics [40].

Enhanced intracellular delivery of KLA peptides and tumor-selective anticancer effects can be achieved using photo-chemo combination liposomes; however, limitations associated with light penetration depth and hypoxic tumor microenvironments remain to be addressed.

### 2.3. Self-Assembled Peptide-Based Nanostructures

Self-assembled peptide-based nanostructures have been developed by exploiting the intrinsic amphiphilic and secondary structural features of the KLA peptide, enabling improved intracellular delivery while maintaining biocompatibility and structural stability.

Molecular self-assembly is a process mediated by weak, non-covalent interactions—hydrogen bonds, electrostatic bonds, hydrophobic bonds, van der Waals interactions, and water-mediated hydrogen bonds [41]. Through appropriate sequence design, peptides can self-assemble into various nanostructures, including nanofibers, nanoparticles and nanotubes, providing control over biomaterials at the molecular level [42]. Self-assembling peptides have gained considerable interest in nanotechnology and biomedicine due to their biomimetic properties, structural design flexibility, excellent biocompatibility and biodegradability, low immunogenicity, and diverse functionality [41].

The structure of pro-apoptotic KLA peptides, with their repeating sequences of positively charged lysine (L) and hydrophobic amino acids, such as leucine (L) and alanine (A), causes these peptides to form α-helical structures [6], making them suitable for self-assembly applications [43]. On the basis of these properties, self-assembly-based delivery strategies have been explored to improve the low cell permeability and stability of KLA peptides.

**Figure 4:**
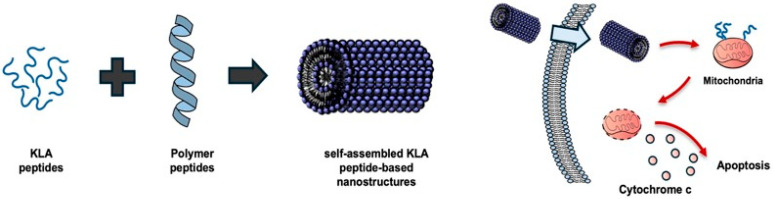
Self-assembled KLA peptide nanostructures. KLA peptides, composed of alternating positively charged and hydrophobic residues, α-helical structures that promote molecular self-assembly into various nanostructures such as nanofibers and nanoparticles. These self-assembled systems improve stability, cellular uptake, and mitochondrial delivery of KLA peptides, leading to mitochondrial membrane disruption and apoptosis in cancer cells. Created using Microsoft PowerPoint (Office 365).

Self-assembly-based nanostructures incorporating cytotoxic peptide KLA peptides have been extensively investigated in the context of cancer therapy (Figure 4). For example, previous reports have demonstrated that a peptide amphiphile containing the KLA peptide with hydrophobic alkyl tails attached to both ends spontaneously self-assembles into supramolecular nanofibers in aqueous solution. This structure showed potent anticancer activity against breast cancer cells through mitochondrial membrane disruption while protecting the peptides from enzymatic degradation [24,44].

Another study reported a strategy for enhancing cancer cell killing by combining the KLA peptide with poly(β-aminoester) to form pH-responsive micelle-like nanoparticles into which the anticancer drug, doxorubicin, is incorporated [45]. Recent studies have focused on strategies for self-assembly in the tumor microenvironment, with polymer–peptide conjugates containing the KLA peptide being reported to form nanoparticles within tumor microenvironments, penetrate deep into solid tumors, and induce cancer cell death [46].

In this system, PT-K-CAA, based on a poly(β-thioester) backbone, was generated by conjugating the pH-sensitive moiety cis-aconitic anhydride (CAA) to the KLA peptide and the cell-penetrating peptide TAT, resulting in a single-chain structure capable of deep penetration into solid tumors [46]. A recent study has demonstrated that the KLA peptide-based nanoassembly RD-KLA-Gffy is capable of inducing apoptosis in central nervous system acute lymphoblastic leukemia cells in vitro and inhibiting tumor growth in vivo [47].

Self-assembling peptide nanostructures provide several advantages, including simplified fabrication, precise molecular design, and high biocompatibility [24,44,48]. In addition, the self-assembly process has been shown to protect KLA peptides from enzymatic degradation and to enhance their pharmacodynamic stability [48].

However, despite these advantages, limitations remain for self-assembled peptide nanostructures. While good biocompatibility and biodegradability are exhibited, the mechanical functional properties are considered to require further optimization. In addition, when used as scaffold materials for in vivo drug delivery, the self-assembly process can be influenced by the physiological environment, and the stability of assembled structures is often insufficient [43].

From a future research perspective, the tumor selectivity and anticancer efficacy of KLA peptides could be further enhanced by simultaneously introducing tumor-targeting motifs (e.g., NGR and RGD peptides) or linkers responsive to different stimuli (e.g., pH, enzymatic, reducing environment) into the KLA peptide-based self-assembly system. The integration of computer-aided peptide design and high-throughput screening technologies may facilitate the development of advanced self-assembling KLA nanostructures.

Overall, in self-assembly-based nanostructures, the KLA peptide can be protected from enzymatic degradation and its therapeutic performance can be enhanced; moreover, incorporation of stimulus-responsive elements or tumor-targeting motifs is expected to further improve anticancer efficacy.

### 2.4. Nanogel-Based Delivery Systems

Nanogel-based delivery systems have been designed to respond to tumor microenvironment associated stimuli, allowing for controlled release and the improved intracellular stabilization of KLA peptides.

Nanogels are hydrogels with a three-dimensional, controllable, porous structure and particle sizes ranging from 20 to 200 nm. Nanogels have gained considerable interest as stable drug carriers in vivo because they maintain their shape without dissolving while maintaining a high-water content. They are particularly suitable for the protection and controlled release of drugs with high side effects or short circulatory half-lives as well as those easily degraded by enzymes, such as anticancer agents, peptides, and proteins [49].

**Figure 5:**
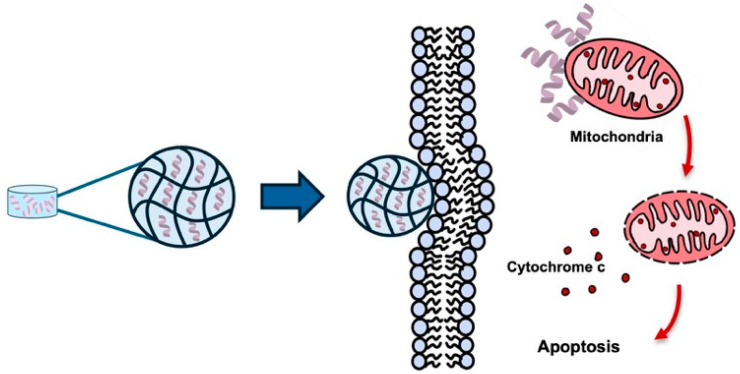
Nanogel-based systems for KLA peptide delivery. Nanogels provide a porous three-dimensional network capable of encapsulating KLA peptides, protecting them from enzymatic degradation and enabling sustained release. Upon exposure to tumor-specific stimuli, the nanogel releases KLA peptides, enhancing intracellular uptake and promoting mitochondrial membrane disruption, inducing apoptosis in cancer cells. Created using Microsoft PowerPoint (Office 365).

One of the reasons for the keen interest in nanogels as drug carriers is their ability to selectively release drugs in tumor tissues in response to the characteristics of the tumor microenvironment (e.g., acidic environment, high reduction potential, ROS) while maintaining drug stability in normal tissues [50]. Encapsulation of the pro-apoptotic KLA peptide within nanogel matrices has been shown to improve intracellular uptake of the KLA peptide (Figure 5).

For example, a novel melittin-RADA_28_ based hydrogel was employed for KLA peptide loading to enhance delivery efficiency in cancer cells [25]. The resulting melittin-RADA_28_-KLA hydrogel, termed MRP, was characterized by a nanofiber structure, sustained-release properties, and reduced hemolytic activity [25]. Enhanced mitochondrial membraned disruption was observed in cancer cells treated with MRP compared with the KLA peptide alone, leading to reduced cell viability [25]. In vivo administration of MRP resulted in approximately 85% inhibition of CT26 tumor growth relative to control groups [25].

In a recent study, a HER2/CD44-targeted hydrogel, although not a nanogel, was constructed incorporating nanocomplexes containing the KLA peptide, survivin siRNA, and Herceptin. This system resulted in enhanced cytotoxicity and uptake in various breast cancer cell lines, indicating that hydrogel-based carriers can also serve as effective platforms for the intracellular delivery of KLA peptides and other biologic anticancer agents [51].

Nanogels have been proposed as an effective platform for the intracellular delivery of pro-apoptotic peptides such as the KLA peptide, enabling enhanced cellular uptake, sustained drug release, and reduced systemic toxicity. Optimization of stimulus-responsive nanogel systems and targeting strategies may improve therapeutic efficacy and broaden the application of KLA peptide-based anticancer agents.

By providing a hydrated three-dimensional network, nanogels facilitate sustained release and efficient intracellular delivery of KLA peptides; further optimization of stimulus-responsiveness and formulation parameters may support their clinical translation.

### 2.5. Homing Domain-Conjugated KLA Peptide

Among the various nano-engineering strategies developed to address the delivery limitations of KLA peptides, homing domain-conjugated KLA peptides have been developed to improve tumor-targeting selectivity and cellular internalization, thereby enhancing the anticancer efficacy of KLA-based therapeutics.

**Figure 6:**
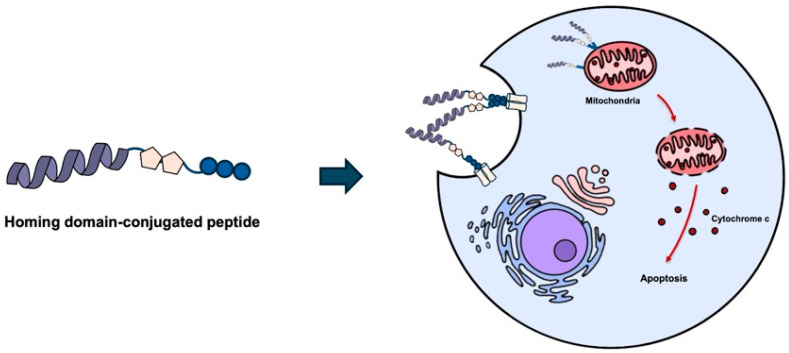
Homing domain-conjugated KLA peptides for tumor targeting. Conjugation of KLA peptides with homing domains, such as cell-penetrating peptides or tumor targeting peptides, enhances cellular uptake and tumor selectivity. Following internalization, KLA peptides translocate to mitochondria and induce mitochondrial membrane disruption, leading to apoptosis. Created using Microsoft PowerPoint (Office 365).

The KLA peptide has been reported to distribute unevenly in vivo and to exhibit limited intracellular delivery efficiency, which reduces anticancer efficacy and may increase toxicity to normal tissues [6,16,17,18,21]. One proposed strategy for overcoming these limitations of the KLA peptide and enhancing its anticancer efficacy is to conjugate it with homing domains, including cell-penetrating peptides (CPPs), that either bind to cancer cell receptors or promote intracellular uptake [25] (Figure 6).

For example, CPPs, including TAT [21] and polyarginine (R7, R8) [52,53,54], consist of amino acid sequences that allow receptor-independent membrane permeation. Combination of CPPs with KLA peptides has been shown to markedly enhance cancer cell death relative to non-modified KLA peptides [21,52,53,54].

Homing domains comprise peptides that selectively interact with specific receptors overexpressed on cancer cell surfaces [21]. iRGD has been reported to bind to αvβ3 and αvβ5 integrins, which are overexpressed in various cancer cells, thereby increasing the targeting and intracellular uptake of KLA peptides into target cancer cells and promoting cancer cell death [55,56,57]. The peptide NVVRQ (termed TMTP1) has been shown to augment the anticancer effect of KLA peptides by targeting metastatic tumor cells [58]. Similarly, the cyclic peptide CNGRC has been demonstrated to improve KLA peptide efficacy by targeting CD13, which is overexpressed in some cancer cells and tumor vascular endothelial cells [59].

Targeting strategies employing homing domains have been associated with enhanced tumor selectivity, reduced normal tissue toxicity, and improved cellular uptake [21]. Recent studies have further expanded homing domain-conjugated KLA peptides, demonstrating enhanced tumor selectivity and anticancer efficacy across various cancer types, including breast cancer, melanoma, and integrin-overexpressing tumors [60,61,62,63].

However, as observed with CPP–nucleic acid complexes, the formation of a protein corona on the surface of the homing domain by various serum components can interfere with cellular uptake and diminish biological activity [64]. Therefore, the design of KLA peptides incorporating homing domains should incorporate strategies to mitigate corona formation, such as corona-resistant coatings and controlled design approaches, rather than relying solely on ligand binding.

Such homing domain-based approaches can reduce nonspecific cytotoxicity in normal tissues and enable tumor-selective delivery; however, their in vivo performance may be influenced by factors such as plasma protein adsorption, necessitating careful molecular and formulation design.

### 2.6. Inorganic-Based Nanoparticles

Inorganic-based nanoparticles, including gold nanoparticles, have been explored to enhance intracellular delivery and mitochondrial targeting of KLA peptides, while enabling the integration of multiple therapeutic modalities.

With the recent emergence of nanomaterials for cancer treatment, the development of novel inorganic nanoparticle-based drug delivery systems for biomedical applications has attracted considerable attention [65]. These nanoparticle-based drug carriers have been proposed to provide higher target specificity and efficacy than conventional small-molecule drugs [65]. By controlling the size, shape, charge, chemical composition, and surface functionalization of nanoparticles, targeted delivery, multifunctionality, and multiple biomolecular interactions can be achieved [22]. Gold nanoparticles, a representative inorganic nanocarrier, exhibit high biocompatibility and can be readily synthesized and characterized, making them suitable as a core material for polypeptide-conjugated nanostructures [66].

**Figure 7:**
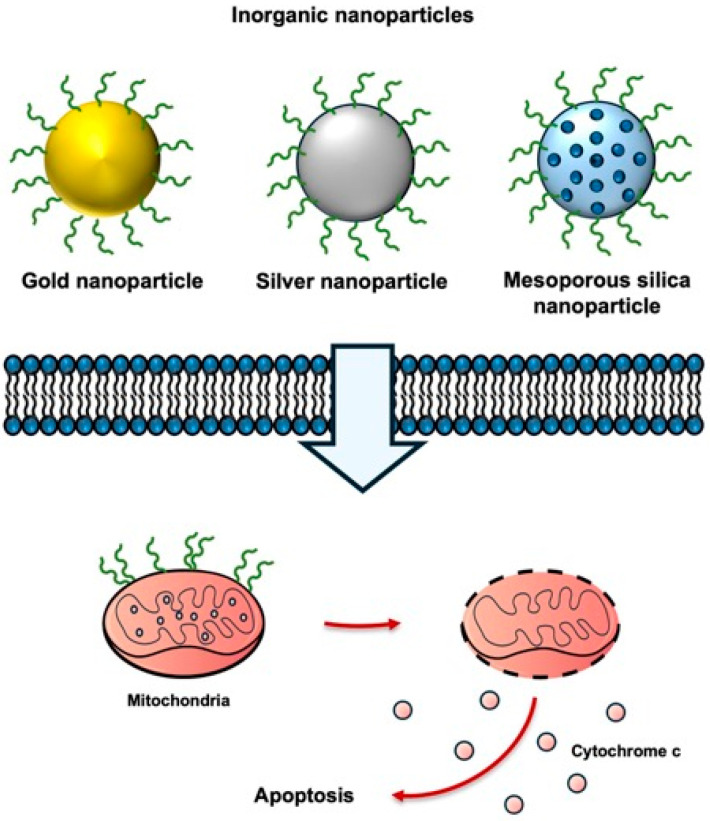
Inorganic nanoparticles for KLA peptide delivery. KLA peptides conjugated to inorganic nanoparticles exhibit enhanced cellular uptake, improved mitochondrial targeting, and increased apoptosis in cancer cells. Created using Microsoft PowerPoint (Office 365).

The physical stability and tunable surface chemical properties of gold nanoparticle-based structures have been demonstrated to enhance the cell-penetrating and mitochondrial-targeting efficiency of the KLA peptide [22], supporting its development as a cancer treatment technology (Figure 7). When immobilized on gold nanoparticles, the KLA peptide exhibits markedly increased anticancer activity compared with treatment using the peptide alone [22].

In a recent study, mitochondria-targeted delivery of KLA peptides was achieved by fluorescent peptidomimetic nanohybrids in U87 glioblastoma cells, resulting in enhanced apoptosis induction and highlighting the potential of inorganic nanoparticle-based systems as multifunctional anticancer platforms [67]. Additionally, copper-based nanoparticles were employed for KLA peptide delivery in 4T1 breast cancer cells, and increased cellular uptake and apoptosis were observed compared with the peptide administered alone [68].

Inorganic nanoparticles have the capability to integrate multiple therapeutic modalities, but several challenges remain [22]. Gold nanoparticles internalized by cells may undergo exocytosis, potentially decreasing therapeutic efficacy [69]. Strategies to enhance intracellular accumulation through surface functionalization have been suggested to mitigate this loss [70]. Efficient endosomal escape of gold nanoparticles after cellular uptake represents a major bottleneck in organelle targeting [22], which can be facilitated by low-energy laser irradiation [71] or surface modification employing the proton sponge effect [72].

Gold nanoparticles are eliminated via the biliary tract and kidneys [73], with additional clearance mediated by the immune system [74]; therefore, consideration of these factors is essential in the design of nanoparticle-based systems. Such improvements in inorganic nanoparticle platforms may contribute to the enhanced anticancer efficacy of KLA peptide-based therapies.

Although inorganic-based nanoparticles enable efficient intracellular delivery and organelle targeting, further optimization is required to address challenges related to intracellular release, endosomal escape, and interactions with the immune system.

### 2.7. Biomimetic Nanocarriers

Biomimetic nanocarriers, including cell membrane-coated nanoparticles and extracellular vesicles, have been investigated to enhance KLA peptide delivery by improving immune evasion, prolonging circulation time, and enabling tumor-targeted delivery.

Nanotechnology-based systems have been developed to provide synthetic drug delivery, protecting drugs, controlling release, and enhancing accumulation at specific tumor sites. Artificial delivery systems exhibit limitations, including inefficient tumor targeting, potential toxicity, and immunosuppressive effects [75]. Biomimetic nanocarriers, particularly cell-derived carriers, have been proposed as an approach to overcome these limitations, due to inherent advantages such as low immunogenicity, prolonged circulation time, and targeting capability [75]. Representative biomimetic nanocarriers include cell membrane-coated nanoparticles [76,77,78] and extracellular vesicles (EVs) [18,79].

**Figure 8:**
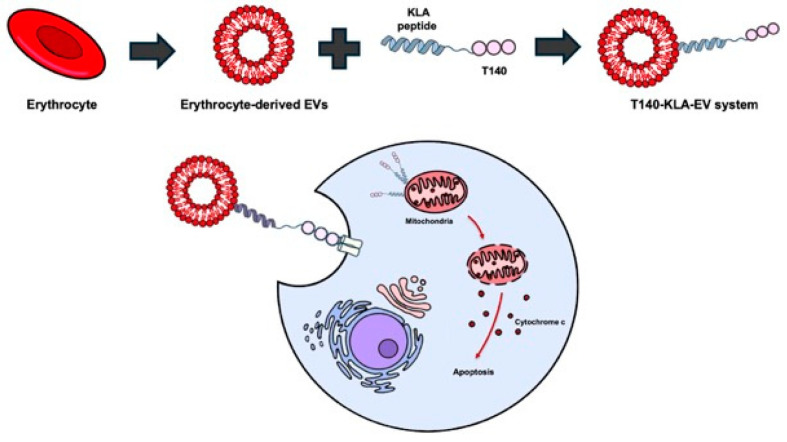
Biomimetic nanocarriers for KLA peptide delivery. Erythrocyte-derived extracellular vesicles loaded with KLA peptides are selectively taken up by cancer cells. Targeting ligands such as T140 facilitate receptor-specific uptake. After internalization, KLA peptides reach the mitochondria and induce apoptosis. Created using Microsoft PowerPoint (Office 365).

Ongoing studies using EVs have sought to overcome the limitations of the KLA peptide, such as poor penetration of the cell membrane and toxicity to non-target cells (Figure 8). These include studies of a dual-functional T140-KLA peptide, designed by linking the CXCR4-targeting peptide, T140, to the C-terminus of the KLA peptide with a GG linker, and binding this peptide to the surface of erythrocyte-derived EVs, yielding a T140–KLA–EV system [18].

In this system, T140 is responsible for target-cell recognition, KLA peptides are responsible for apoptosis induction, and EVs are responsible for intracellular delivery efficiency, circulation stability, and enhanced biocompatibility. In another study [80], methotrexate-loaded EVs were functionalized with a blood–brain barrier (BBB)-penetrating LDL peptide and KLA peptide for the treatment of glioblastoma. The LDL peptide was reported to promote EV passage through the BBB and selective uptake into tumor cells, whereas the KLA peptide was able to induce cancer cell apoptosis. To date, cell membrane-coated nanoparticles have not been reported for KLA peptide delivery.

Biomimetic systems present challenges, including difficulties in large-scale production and structural stability [80,81]. Strategies for uniform manufacturing, reduced immune responses, and optimized combination therapy platforms have been proposed to address these challenges. Such optimization may facilitate the development of effective KLA peptide-based anticancer therapeutics.

While biomimetic nanocarriers reduce non-target cell toxicity and enable efficient intracellular delivery of KLA peptides, challenges remain in large-scale production, structural stability, and optimization of combination therapy strategies.

The key advantages and limitations of the various nano-engineering-based delivery platforms for KLA peptides are summarized in Table 1. This overview provides a systematic comparison, highlighting trade-offs and guiding the design of next-generation KLA peptide therapeutics.

Various nano-engineering strategies have been developed for the delivery of KLA peptides. Examples of these strategies, including their peptide structures and the target cancer types, are summarized in Table 2.

## 3. Synergy and Combination Therapy Strategies

The various nano-engineering-based KLA peptide-delivery systems highlighted in Section 2 have been developed to improve low cell permeability and poor tumor selectivity of the KLA peptide. Advances in these delivery platforms have laid the foundation for more precisely controlling KLA peptide-based anticancer strategies. In particular, synergistic combination-treatment strategies are under investigation to maximize the anticancer effects of the KLA peptide. Although most studies remain at the preclinical stage, a number of combination strategies may contribute to the future development of technologies aimed at enhancing cancer treatment efficacy.

First, the KLA peptide-liposome system and pH-responsive delivery system introduced in Section 2 are expected to play a key role in combination strategies. For example, KLA peptide-modified liposomes co-loaded with 5-fluorouracil and paclitaxel demonstrated complementary action, enhancing anticancer effects in triple-negative breast cancer (TNBC) [82]. Similarly, co-loading pH-responsive nanosystems described in Section 2.1 with KLA peptide and DTX was shown to simultaneously induce mitochondrial disruption and inhibit cell-cycle progression, the maximizing synergistic effect [7]. These findings indicate that single stimulus-based delivery systems can serve as effective platforms for combination therapy.

In addition, a combination strategy with PDT—an extension of the photo-responsive system introduced in Section 2—is also establishing itself as an important research trend. In particular, in the case of liposomes co-loaded with the KLA peptide and chlorin e6, it has been reported that ^1^O_2_ generated after light irradiation effectively disrupts endosomal and lysosomal membranes, thereby increasing accessibility of the KLA peptide to mitochondria and inducing a potent cancer cell-killing effect [37]. This is a representative example of how the photo-chemo combination-based delivery system described in Section 2 constitutes a practical combination strategy for enhancing anticancer efficacy.

Combination strategies involving radiotherapy have also been investigated. The CPP-based cell-penetration technology discussed in Section 2 can enhance the delivery efficiency of the KLA peptide even to radioresistant cancer cells. It has been reported that intratumoral injection of the KLA peptide using a CPP followed by gamma irradiation results in a higher level of cancer cell death compared with radiotherapy alone due to a combination of mitochondrial and DNA damage [15].

The homing domain-based cancer cell-targeting strategy presented in Section 2 has recently been extended to include combination strategies with immunotherapy. For example, combining melittin with the KLA peptide was reported to selectively kill M2-type tumor-associated macrophages, which drive the immunosuppressive environment [83]. This is significant in that it suggests that immune modulation of the tumor microenvironment can synergistically enhance the efficacy of anticancer or immunotherapy agents.

The various KLA peptide delivery technologies discussed in Section 2 provide the foundation for combination and synergistic therapeutic strategies incorporating conventional cancer treatments. Each platform demonstrates complementary effects when combined with chemotherapy, photodynamic therapy, radiotherapy, or immunotherapy.

Table 3 provides a concise comparative overview, highlighting how structural modifications influence mechanistic behavior and therapeutic outcomes, thereby guiding the rational design of combination strategies.

However, most of these studies remain preclinical, at the cellular, or mouse model level. Furthermore, practical challenges remain, including long-term toxicity, immunogenicity, administration methods, and large-scale production. Therefore, future development of KLA peptide-based cancer therapeutics requires not only the demonstrated efficacy of combination strategies, but also improvements in the stability and functionality of the delivery system.

## 4. Clinical Translation Challenges and Critical Limitations

Although KLA peptide-based nanotherapeutics have shown efficacy in preclinical studies, further research is required for clinical translation.

### 4.1. Structural and Manufacturing Challenges

Liposome-based KLA peptide nanotherapeutic systems encounter substantial structural and process-related challenges during development and commercialization. These include complexities associated with particle characterization, stability, reproducibility of manufacturing, analytical method validation, and scalability [84]. Similar obstacles can also arise with other self-assembled peptides, nanogels, inorganic nanoparticles, and biomimetic carriers, particularly because of material-specific issues such as in vivo stability, immune activation, and long-term accumulation.

### 4.2. Tumor Microenvironment Heterogeneity

The intrinsic heterogeneity of the tumor microenvironment can markedly reduce the precision and efficiency of targeted delivery systems. Because human tumors exhibit extensive genetic and phenotypic variability, pH-responsive or receptor-targeted carriers that show promising outcomes in preclinical studies may not translate effectively to clinical applications [85]. This limitation is especially relevant for KLA peptide systems used in combination with photodynamic therapy, where fluctuations in the tumor microenvironment may compromise the activity of photosensitizers. These challenges underscore the need for delivery strategies that are adaptable to microenvironmental variations [38,39].

### 4.3. Safety, Toxicity, and Off-Target Effects

Uncertainty regarding in vivo stability and long-term toxicity remain significant barriers to clinical translation [86]. Variations in the biodistribution, immune responses, and accumulation profiles of nanomaterials or surface-modified carriers can lead to unanticipated adverse effects. Furthermore, the intrinsic positive charge of the KLA peptide can induce off-target cytotoxicity in normal tissues. Therefore, a comprehensive assessment of chronic toxicity, immunogenicity, metabolic processing, and clearance pathways is imperative for safe clinical application.

For CPP-conjugated anticancer peptides, targeting ability, cellular uptake, and overall biological activity may be diminished due to adsorption to plasma proteins upon entering the bloodstream [65]. Therefore, when developing anticancer agents that combine KLA peptides with homing peptides or CPPs, it is essential to incorporate strategies that enhance their stability in the bloodstream and control the reduction in their delivery efficiency.

### 4.4. Limitations of Preclinical Models

Most studies remain limited to short-term preclinical models such as mice, which do not recapitulate the complexity of the human tumor microenvironment. Another significant limitation is insufficient characterization of clinically relevant factors, including pharmacokinetics, pharmacodynamics, cumulative toxicity associated with repeated dosing, and conditions involving combination therapy.

### 4.5. Regulatory Considerations for KLA Peptide-Based Nanotherapeutics

The FDA and EMA require that nanomedicines possess consistent quality and reproducible manufacturing processes and be subjected to rigorous evaluations to ensure stability and efficacy. In KLA peptide-based systems, particle size, surface properties, and encapsulation efficiency must be controlled, and stability should be verified using validated analytical methods. As no specific regulations exist for KLA peptides, general nanomedicine guidelines are to be followed.

Stability assessment should include long-term effects, such as biodistribution, immune responses, and toxicity upon repeated dosing. CPP or homing peptide conjugation, tumor microenvironment heterogeneity, stimulus-responsive designs, and combination therapies may be subject to additional regulatory review.

### 4.6. Mechanistic Rationale for KLA Peptide-Based Combination Therapy

Combination therapy involving KLA peptides is mechanistically supported by their unique mode of action, in which apoptosis is induced through mitochondrial membrane disruption in cancer cells. This mitochondrial targeting mechanism is complementary to conventional anticancer therapies that primarily rely on DNA damage induction, oxidative stress generation, or immune modulation. When combined with chemotherapeutic agents, mitochondrial dysfunction induced by KLA peptides can lower the apoptotic threshold of cancer cells, thereby enhancing drug-mediated cytotoxicity.

In the context of photodynamic therapy, reactive oxygen species generated upon light irradiation can increase mitochondrial accessibility, resulting in the potentiation of KLA peptide-mediated cytotoxic effects in cancer cells. Similarly, combination with radiotherapy may concurrently induce mitochondrial dysfunction and DNA damage, leading to synergistic anticancer activity. In addition, apoptosis triggered by KLA peptides may contribute to immunogenic cell death, providing a mechanistic basis for combination strategies involving immunotherapy. Collectively, these mechanistic considerations indicate that KLA peptide-based combination therapies represent a clinically rational approach based on complementary modes of action rather than simple additive effects.

## 5. Conclusions and Future Directions

The KLA peptide, a mitochondria-targeting apoptosis inducer, exhibits therapeutic potential. However, the KLA peptide exhibits poor cellular uptake and lacks targeting specificity, limiting its clinical potential in cancer therapy. Recent advances in various nano-engineering-based delivery technologies have provided significant breakthroughs that could overcome these limitations. 

As summarized in this review, pH-responsive nanosystems, photo-chemo combination liposome system, self-assembled peptide nanostructures, nanogels, homing domain conjugation technology, inorganic nanoparticles, and biomimetic nanocarriers have significantly improved the stability, cellular-uptake efficiency, mitochondrial accessibility, and tumor specificity of KLA peptides. 

Although KLA-peptide therapy combined with nanocarriers holds considerable therapeutic potential, several challenges must be resolved before clinical application can be realized. These include standardizing manufacturing processes, ensuring long-term stability; overcoming challenges associated with tumor microenvironment heterogeneity, particularly as it relates to targeting efficiency; verifying in vivo stability, long-term toxicity and immune responses; and addressing protein corona formation in homing peptide or CPP-conjugated carriers. 

In addition, most current research remains in the small animal-based preclinical stage; future research must include clinically meaningful pharmacokinetic and pharmacodynamic analyses and repeated-dose stability assessments, including in advanced large-animal models. Systematically overcoming these barriers will be essential for accelerating the clinical translation of KLA peptide-based anticancer therapeutics. 

Looking forward, polypeptide-based nanoparticles represent a promising and flexible strategy for next-generation KLA peptide delivery. Such platforms may enable the efficient encapsulation or covalent conjugation of KLA peptides, stimulus-responsive and controlled release, and improved pharmacokinetic stability, while enhancing intracellular and mitochondrial accessibility. Integration of stimulus-responsive design, tumor-specific targeting motifs, biomimetic delivery concepts, and advanced peptide engineering approaches, including computational design and high-throughput screening, is expected to further accelerate the development of optimized KLA peptide-based nanotherapeutics. 

In summary, nanotherapy using KLA peptides is a promising anticancer strategy with high potential. Going forward, the development of next-generation KLA-based nanotherapy systems that integrate smart stimulus-responsive design, tumor-specific targeting technology, biomimetic platforms, computational peptide design, and high-throughput screening technologies will be crucial. Stepwise resolution of these biological and technological barriers may enhance the clinical feasibility of KLA peptide-based anticancer therapy.

## Figures and Tables

**Table 1:** Comparative overview of nano-engineering-based KLA peptide delivery systems, including key advantages, major limitations, and translational challenges.

Delivery System	Key Advantages	Major Limitations	Translational Challenges	References
pH-responsive nanosystems	Tumor-selective release, reduced off-target toxicity	Synthetic complexity	Scalability	[7,27,29]
Photo-chemo combination liposomes	Strong synergistic ROS/apoptosis	Light penetration, hypoxia dependence	Clinical device integration	[37,38,39,40]
Self-assembled peptide-based nanostructures	High biocompatibility, protease resistance	Structural instability in vivo	Batch consistency	[24,43,44,45,46,47,48]
Nanogel-based delivery systems	Sustained release, reduced systemic toxicity	Formulation optimization	Manufacturing standardization	[25,49,50]
Homing domain-conjugated KLA peptide	Enhanced targeting specificity	Protein corona, off-target uptake	In vivo robustness	[50,64]
Inorganic-based nanoparticles	Multifunctionality, stable surface chemistry	Immune clearance, endosomal escape	Long-term safety	[22,69,70,71,72,73]
Biomimetic nanocarriers	Immune evasion, prolonged circulation	Scale-up difficulty	Regulatory complexity	[18,79,80,81]

**Table 2:** Examples of KLA-based peptide structures and their target cancer types. The table summarizes the type of delivery platform, the KLA peptide form, the cancer type it was applied to, key features, and the references.

Delivery System	KLA Peptide Form	Target Cancer Type	Key Features/Notes	References
pH-responsive nanosystems	CGKRD(KLAKLAK)_2_ D-(KLAKLAK)_2_	Breast cancer Melanoma	pH-sensitive release, mitochondrial targeting, co-delivery with docetaxel	[7] [27]
Photo-chemo combination liposomes	D-(KLAKLAK)_2_ in Lipo[Pep,Ce6]	Oral squamous cell carcinoma	PDT + KLA synergy, ROS-mediated mitochondrial disruption	[37]
Self-assembled peptide-based nanostructures	KLA peptide amphiphiles PT-K-CAA RD-KLA-Gffy	Breast cancer Melanoma CNS leukemia	Self-assembly protects from enzymatic degradation, pH responsive, deep tumor penetration	[24,44] [46] [47]
Nanogel-based delivery systems	Melittin-RADA_28_-KLA hydrogel ALPR-KLA	CT26 tumor cells Breast cancer	Sustained release, enhanced mitochondrial disruption	[25] [51]
Homing domain-conjugated KLA peptide	TAT-KLA R7/R8-KLA iRGD-KLA TMTP1-KLA CNGRC-KLA	Breast cancer Melanoma Metastatic tumors Highly metastatic cancer Integrin-overexpressing tumors	Tumor-targeted delivery, enhanced cellular uptake	[21] [52] [55] [58] [59]
Inorganic-based nanoparticles	KLA on gold KLA on AgInS_2_ KLA on copper nanoparticles	Cervical cancer Glioblastoma 4T1 breast cancer	Enhanced mitochondrial delivery, multifunctional platform	[22] [67] [68]
Biomimetic nanocarriers	T140-KLA-EV LDL-KLA-EV	Lung cancer Glioblastoma	Immune evasion, BBB penetration, apoptosis induction	[18] [80]

**Table 3:** Summary of structural modification. Mechanistic effects and therapeutic index of KLA peptide delivery platforms.

Delivery System	Structural Modifications	Mechanistic Effect	Impact of Therapeutic Index
pH-responsive nanosystems	pH-sensitive polymers, micelle formation	Enhanced mitochondrial access, selective tumor uptake	Increased efficacy, reduced off-target toxicity
Photo-chemo combination liposomes	Photosensitizer incorporation, KLA peptide co-loading	ROS-mediated endosomal escape, mitochondrial disruption	Synergistic cytotoxicity, improved tumor selectivity
Self-assembled peptide-based nanostructures	Amphiphilic peptide design, α-helical self-assembly	Stabilized peptide, efficient intracellular delivery	Improved apoptosis induction, reduced enzymatic degradation
Nanogel-based delivery systems	Hydrated 3D networks, stimulus-responsive crosslinking	Controlled release, sustained intracellular presence	Enhanced efficacy, lower systemic toxicity
Homing domain-conjugated KLA peptide	CPP or targeting peptide conjugation	Receptor-mediated uptake, improved tumor targeting	Increased tumor selectivity, reduced normal tissue toxicity
Inorganic-based nanoparticles	Gold nanoparticle conjugation	Enhanced mitochondrial targeting, multifunctional delivery	Potentiated anticancer effect, integration with other therapies
Biomimetic nanocarriers	Cell membrane coating, extracellular vesicle loading	Immune evasion, prolonged circulation, targeted delivery	Improved therapeutic window, efficient intracellular delivery

## Data Availability

No new data were created or analyzed in this study. Data sharing is not applicable to this article.
